# Impacts of Hypoxia on Osteoclast Formation and Activity: Systematic Review

**DOI:** 10.3390/ijms221810146

**Published:** 2021-09-20

**Authors:** Jen Kit Tan, Nur Shukriyah Mohamad Hazir, Ekram Alias

**Affiliations:** 1 Department of Biochemistry, Faculty of Medicine, Pusat Perubatan Universiti Kebangsaan Malaysia, Bandar Tun Razak, Kuala Lumpur 56000, Malaysia; jenkittan@ukm.edu.my (J.K.T.); nurshukriyah@unikl.edu.my (N.S.M.H.); 2 Clinical Laboratory Section, Institute of Medical Science Technology, Universiti Kuala Lumpur, Taman Kajang Sentral, Kajang 43000, Malaysia

**Keywords:** hypoxia, oxygen, osteoclast, bone loss, systematic review

## Abstract

Hypoxia is evident in several bone diseases which are characterized by excessive bone resorption by osteoclasts, the bone-resorbing cells. The effects of hypoxia on osteoclast formation and activities are widely studied but remain inconclusive. This systematic review discusses the studies reporting the effect of hypoxia on osteoclast differentiation and activity. A literature search for relevant studies was conducted through SCOPUS and PUBMED MEDLINE search engines. The inclusion criteria were original research articles presenting data demonstrating the effect of hypoxia or low oxygen on osteoclast formation and activity. A total of 286 studies were identified from the search, whereby 20 studies were included in this review, consisting of four in vivo studies and 16 in vitro studies. In total, 12 out of 14 studies reporting the effect of hypoxia on osteoclast activity indicated higher bone resorption under hypoxic conditions, 14 studies reported that hypoxia resulted in more osteoclasts, one study found that the number remained unchanged, and five studies indicated that the number decreased. In summary, examination of the relevant literature suggests differences in findings between studies, hence the impact of hypoxia on osteoclasts remains debatable, even though there is more evidence to suggest it promotes osteoclast differentiation and activity.

## 1. Introduction

Pathological bone loss, like osteoporosis and rheumatoid arthritis, is often caused by excessive bone erosion [1,2,3]. This excessive bone loss is usually caused by either an increase in bone resorption carried out by osteoclasts or a suppression of osteoblasts to form new bone. The increase in bone resorption could have resulted from the increased osteoclast differentiation, as evident from a high number of osteoclasts and elevated expression of the cell markers seen in tissues of bone loss diseases like osteoporosis [1], rheumatoid arthritis [2,3,4], and peri-implant osteolysis [5].

Hypoxia is evident in several bone pathologies such as rheumatoid arthritis [1], pathological fracture [2], primary bone tumors [3], and cancer metastases [4], as well as other conditions such as inflammation, ischemia, and infection (less than 1%) [5]. Moreover, previous studies also reported pathological bone loss often observed at sites with a low partial pressure of oxygen (pO_2_) [6,7]. Oxygen (O_2_) in tissues is generally between 5–12% [7], therefore hypoxia or low O_2_ tension in tissues may represent disease states.

Cells under hypoxic conditions are usually characterized by high expression of hypoxia-inducible factor (HIF) proteins, commonly either HIF-1α or HIF-2α, which are also the transcription factors that mediate hypoxia-related pathways. In the presence of O_2_, HIF-α is constitutively hydroxylated to be destined for proteasomal degradation. However, this does not occur under hypoxic conditions, leaving HIF-α to remain stable in the cell and able to dimerize with HIF-β then bind to hypoxia-response element and initiate transcription of HIF target genes [8].

Findings that suggest an association between hypoxia and the aforementioned bone diseases have prompted studies investigating the impact of hypoxia on osteoblasts and osteoclasts which are actively involved in bone metabolism. Hypoxia has been found to suppress bone formation by inhibiting proliferation and differentiation of osteoblasts [9] as well as altering the bone mineralization capability of osteoblasts [10]. On the other hand, an accumulating number of studies indicate an increase in bone resorption due to an increase in osteoclast differentiation and activity following hypoxia.

Osteoclasts are giant multinucleated cells specialized in resorbing the bone. The differentiation of pre-osteoclasts, which are from the monocyte/macrophage lineage, into mature cells capable of resorbing bone, involves the activation of receptor activator of nuclear factor-kappa B (RANK) upon its ligand (RANKL) binding [11]. This leads to a cascade of intracellular events such as activation of nuclear factor-кβ (NFкβ), mitogen-activated protein kinase (MAPK), phosphoinositide 3-kinase /protein kinase B (PI3K-Akt), and calcium signaling [12] further followed by expression and activation of nuclear factor of activated T-cells 1 (NFATc1), the key transcriptional factor for osteoclastogenesis [13]. In turn, NFATc1 will stimulate the expression of several osteoclast markers like cathepsin K, calcitonin receptor, and integrin-β3 [14,15,16]. Besides RANKL, macrophage colony-stimulating factor (MCSF) is another cytokine that plays an important role in promoting osteoclast differentiation (or also known as osteoclastogenesis) [17]. This cytokine is believed to have a role in regulating the RANK receptor activation [18].

The cellular metabolism in osteoclasts, particularly during osteoclastogenesis, received much attention and has been widely discussed lately. The abundance of mitochondria in osteoclasts, which is believed to suit the purpose of multinucleation process during osteoclastogenesis, may suggest the importance of aerobic respiration in generating a high amount of energy in osteoclast differentiation or function [19]. Thus, considering the aerobic mitochondrial respiration and oxidative phosphorylation are diminished in low oxygen conditions, it is interesting to examine how hypoxia impacts osteoclastogenesis.

This systematic review serves the purpose of examining the evidence indicating the effect of hypoxia on the differentiation of osteoclasts and their bone-resorbing activity. From this systematic review, the opinion on the impact of hypoxia on osteoclasts could be consolidated. This study will also provide the basis for using hypoxia-related pathways as alternative therapeutic targets for pathological bone loss.

## 2. Results

### 2.1. Search Results

**Figure 1:**
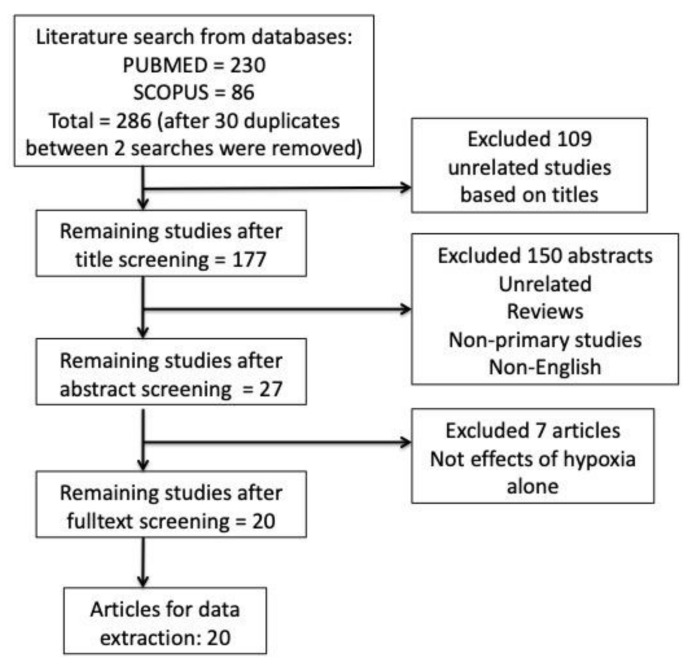
Flowchart of the article selection strategies for this systematic review.

The flow chart of the article selection process is shown in Figure 1. A total of 316 articles were found from the two search databases. After a brief screening based on the titles of articles, 30 duplicates and 109 unrelated studies were removed. Then, 150 articles were excluded after the abstract screening, which was a more thorough process, as they were unrelated, reviews, or not published in English. Upon full-text screening, seven articles were excluded because they did not have data reporting the effect of hypoxia alone. All remaining 20 articles were analyzed for data extraction.

### 2.2. Study Characteristics

The details of all the selected studies were summarized in Tables S1–S4. Those articles were published between the years 2003 to 2020, which consisted of 16 in vitro and four in vivo studies. Various types of osteoclasts were used in the in vitro studies, ranging from the peripheral blood mononuclear cells (PBMCs)-derived osteoclasts to osteoclast-like giant cells from giant cell tumor of bone, RAW264.7 cell line, osteoclasts differentiated from human or murine bone marrow cells (BMCs), and mature osteoclasts from a rat. Five in vitro studies reviewed here used human PBMC-derived osteoclasts differentiated from CD14-positive mononuclear cells isolated from the blood [4,5,6,7,8] (Table S1). Muzylak et al. isolated the PBMCs from cat blood [9]. In all studies on PBMC-derived osteoclasts, cells were cultured in the presence of exogenous RANKL and MCSF. TheRAW264.7 cell line (murine macrophages) used in six studies was cultured with or without RANKL and MCSF [10,11,12,13,14] (Table S2). Some studies used BMCs isolated from human bones of patients who underwent total hip arthroplasty for arthritis [15] or from bones of mice [16,17,18,19] (Table S3). These isolated cells were cultured with RANKL and MCSF to promote the differentiation of osteoclasts. A study also used osteoclast-like giant cells from human giant cell tumor of bone, beside a PBMC-derived osteoclast model [8] (Table S1). Besides RAW264.7-derived osteoclasts, Arnett and coworkers (2003) also studied the impact of hypoxia on osteoclasts that were isolated directly from the bones of rats [19] (Table S3) as a model of mature osteoclast. Rats and mice were used in the in vivo studies (Table S4).

The condition of hypoxia varied in terms of oxygen (O_2_) percentage and exposure duration. The concentration of O_2_ used to represent the hypoxic condition in vitro ranged from 12% [19] to as low as 0% [18]. The duration of hypoxic exposure ranged from 2 h to 21 days. There were two in vitro studies that alternated the exposure duration between hypoxia and normoxia. Knowles et al. [8] and Nomura et al. [15] performed scheduled cycles of hypoxia and normoxia for 7–21 days. Normoxia or reoxygenation was defined at 20–21% O_2_. The hypoxia setting in the in vivo studies appeared more complex than in the in vitro studies since animals could not live under hypoxia. In the only in vivo study involving rats, they were exposed to intermittent hypobaric hypoxia [20]. Wang et al. used an intermittent high-altitude (5000 m) imitation on normal or ovariectomized rats of Sprague-Dawley rats to resemble hypobaric hypoxia [20]. The partial pressure of oxygen, pO_2_, that was equivalent to the experimental 5000 m high-altitude as used in the study was 84 mmHg [21], which represented approximately 47% reduction in the O_2_ content. Using a similar approach to Wang et al. [20], Durand et al. [22] also placed the studied mice into a hypobaric chamber to reduce the O_2_ content in air from 21% to 10% and assessed the impact of continuous hypoxia (10% O_2_) for four days on the tested animals [22]. Dalle Carbonare et al. [23] used transgenic mice from a humanized sickle cell disease model and healthy controls to study the effect of hypoxia/ reoxygenation stress. In their work, the mice had hypoxia stress (8% O_2_) for 10 h followed by 3 h of reoxygenation. The induction of hypoxia condition to tissues in a study by Takemori et al. (2019) was unique compared to the others since it did not involve manipulating O_2_ content in the air but through applying CO_2_ hydrogel to corresponding tissue [24]. The number of animals used per group in these four studies ranged from six to 10.

All 16 in vitro studies reviewed here assessed the impact of hypoxia on osteoclast differentiation. While a majority of those in vitro studies used tartrate-resistant acid phosphatase (TRAP) staining as a method for assessing osteoclast formation, some also used phalloidin staining [10] and immunostaining with other osteoclast markers such as vibronectin receptor (VNR) [6,8]. Data on gene or protein expression of osteoclast markers like cathepsin K, calcitonin receptor, and TRAP as observed in a number of the in vitro studies [4,5,10,11,12,14,16,17] was also revealed to be associated with osteoclast formation. The impact of hypoxia on osteoclast bone resorbing activity was studied in 10 in vitro studies, in which resorption was assessed from toluidine blue staining [6,7,8,10,17,19], bisphosphonate fluorescence [4], biotin-conjugated wheat germ agglutination lectin staining [9], and Corning Osteo assay [5,16]. Osteoclast activity described in these articles refers to the extent of bone being resorbed by the osteoclasts [25,26].

In two in vivo studies described in this review, the effect of hypoxia on the bone resorption was assessed through static bone histomorphometric parameters: eroded surface/total bone surface (ES/BS), osteoclast number/total bone surface (Oc. N/BS), and the number of osteoclasts/total area (N. Oc/TA) [22,23]. On the other hand, osteoclast formation following hypoxia was also assessed based on the number of osteoclasts in the femur following TRAP [20,24]. In determining the impact of hypoxia on bone resorption systemically, the levels of bone resorption marker, C-telopeptide of type-1 collagen (CTX-I), in serum was measured [20,23]. The study by Takemori et al. also carried out measurement using micro-computed tomography (micro-CT) for bone structural analysis [24].

### 2.3. Effects of Hypoxia on Formation and Activity of Osteoclasts In Vitro

All selected articles compared the number of osteoclasts formed under hypoxic and normoxic conditions (Tables S1–S3). Among the 16 in vitro studies, five studies showed that hypoxia enhanced osteoclast differentiation and bone resorption in the presence of RANKL and MCSF [5,7,10,16,19]. Other studies that assessed osteoclast differentiation but did not look at osteoclast activity in vitro also demonstrated more osteoclasts formed in hypoxia [11,12,13,15,18]. Even though in the in vitro study by Srinivasan et al. [14] hypoxia seemed to promote osteoclast formation seen from the number of TRAP-positive cells and gene expression levels of osteoclast markers, no statistically significant difference between treatment was indicated.

All studies that measured the gene and protein expression of osteoclast markers reported that the data were consistent with their observation on the changes in the total number of osteoclasts formed in hypoxia. The increase in the number of osteoclasts following hypoxia seen in a number of these studies was accompanied by an elevation in the gene and protein expression of osteoclast markers, including cathepsin K, MMP9, β3-integrin, calcitonin receptor, and TRAP [5,10,11,12,16]. On the other hand, the reduction in osteoclastogenesis following hypoxia reported in two studies [4,17] was consistent with the decrease in gene and protein expression of carbonic anhydrase II, cathepsin K, β3-integrin, TRAP, RANK, and DC-STAMP.

Other than the study by Ma et al. [17] and Gorissen et al. [4], findings from all other articles reporting the impact of hypoxia on osteoclast activity, either in vitro or in vivo models [5,6,7,9,10,16,19,20,23], indicated that the total resorption was higher in the hypoxic conditions. Among these in vitro studies, some found that hypoxia increased osteoclast bone-resorbing activity, but did not promote osteoclast differentiation [6,8,9]. On the other hand, Gorissen et al. (2018) found that hypoxia decreased the number of multinucleated osteoclasts being formed by delaying the cell differentiation process. Meanwhile, the study by Ma et al. [17] found that hypoxia (1% O_2_) reduced both osteoclast formation and activity in vitro. Hypoxia could increase total bone resorption either by promoting osteoclastogenesis or enhancing the osteoclast capacity for resorbing bone. Findings by Muzylak et al. [9] suggest that the increase in bone resorption, despite a reduction of viable osteoclast, could be due to an increase in osteoclast size as promoted by hypoxia. However, this might not be in agreement with the finding by Gorissen et al. [4] which demonstrated that hypoxia suppressed cell multinucleation during osteoclast differentiation.

Studies using models of mature osteoclasts such as human osteoclast-like giant cells of human giant cell of tumor bone [8] and osteoclasts isolated from rat bones [19] suggested that hypoxia stimulated the activity of osteoclasts at the expense of reducing the lifespan of osteoclasts. In Gorissen et al. [4], osteoclasts formed in normoxia showed senescence as characterized by higher expression of p16, p21, and senescence-associated β-galactosidase, but not in hypoxia. Delayed osteoclastogenesis in hypoxia conditions may be due to cell death caused by low oxygen at 5%.

Data from studies by Utting et al. [7] and Murata et al. [5] suggested that the hypoxia-induced osteoclast formation could only take place in the presence of RANKL. Findings from Srinivasan et al. [14] and Nomura et al. [15], together with Murata et al. [5], indicated that the addition of exogenous RANKL in the cell culture gave an additive effect in promoting the hypoxia-induced osteoclastogenesis in a dose-dependent manner. However, data in the study by Ma et al. [17] indicated that hypoxia suppressed osteoclast differentiation even in the presence of exogenous RANKL and MCSF.

### 2.4. Effects of Hypoxia on Formation and Activity of Osteoclasts In Vivo

Wang et al. demonstrated that reduction in O_2_ level in air accelerated bone loss in ovariectomized rats by enhancing the formation of osteoclasts (as indicated from TRAP staining), while having no effect on normal rats [20]. This increase in the number of osteoclasts being formed observed in ovariectomized rats following exposure to a low concentration of O_2_ was accompanied by an elevation in the level of serum bone resorption marker CTX-1 in the same group of rats.

Dalle Carbonare et al. showed that hypoxia (8% O_2_ for 10 h) followed by reoxygenation (21% O_2_ for 3 h) had a profound effect in promoting osteoclastogenesis (as characterized by increased osteoclast number and elevated mRNA expression of osteoclast markers RANK and cathepsin K) in the transgenic mouse model of sickle cell disease used [23]. This increase in osteoclasts was accompanied by more bone erosion (indicated by more bone eroded surface and a higher level of CTX) observed in the corresponding animals [23]. Similarly, the study by Durand et al. [22] also demonstrated that hypoxia (FiO_2_ = 10%) for four days in normal C57BL/6J mice doubled the number of osteoclasts on the femurs despite inducing no bone loss (Table S4). The authors speculate that the hypoxic exposure was rather too short to observe any change in the bone structure.

It was interesting to note that hypoxia on tissues in vivo through the transcutaneous application of CO_2_ hydrogel demonstrated by Takemori and coworkers [24], on the other hand, resulted in a reduction in numbers of osteoclasts being formed in the adjacent bone. It was an unexpected finding since in vitro data from the same study indicated that the cancer cells or tissues on which the CO_2_ hydrogel was applied had increased mRNA expression of osteoclast differentiation factors RANKL [24].

## 3. Discussion

The impact of hypoxia on bone remodeling and health, or more specifically on osteoclasts, has been widely studied, as indicated by numerous studies obtained from the search. While the impacts of hypoxia on osteoclasts have been studied for years, to the best of our knowledge, this article is the first systematic review on this topic. This systematic review included articles that presented data reporting the effect of hypoxia alone on osteoclast formation and/or activity. The use of various study models for osteoclasts in the studies reviewed here, including the common and well-established in vitro models of RAW264.7, BMC, and PBMC-derived osteoclasts, allowed us to provide evidence-based opinion on the effect of hypoxia on osteoclasts and subsequently towards bone erosion.

During the selection of articles, studies in which hypoxia was represented in the form of overexpression of HIF-1α or HIF-2α only were not included in the review. This was because there was evidence to suggest that increased HIF-1α expression or stabilization does not necessarily indicate hypoxia. A study by Hulley et al. [6] found mRNA and protein expression of HIF-1α increased during the differentiation of PBMC-derived osteoclasts, even in normoxic conditions. On the other hand, silencing or inducing HIF-1α mRNA expression did not give a similar response to osteoclast formation and activity as hypoxia did [6]. In addition, there is evidence to indicate that the level of HIF-1α could be enhanced by non-hypoxic stimuli such as lipopolysaccharides and thrombin [27]. Furthermore, elevation in HIF-1α expression in osteoclasts had been observed in ovariectomized animals in normoxia [28]. Since HIF is the abbreviation for hypoxia-induced factor, the inclusion of the word “hypoxia” in the literature search could be the reason as to why many articles studying either HIF-1α or HIF-2α function alone without low oxygen tension appeared in the search list.

The hypoxic conditions used by the selected studies reviewed here are summarized in Tables S1–S4. There is no standardized level of oxygen that defines hypoxia in in vitro studies. A number of the reviewed studies [4,13,14] regarded 5% O_2_ as considerably hypoxic in vitro compared to the normoxic conditions. Other studies [5,6,10,16] used 2% O_2_ to represent the in vitro hypoxic condition, while others applied multiple O_2_ concentrations in their experiments [8,9,15,18,19,29]. There are also studies [11,12,17] that employed extremely low concentrations of O_2_ (1% or lower) for the hypoxia treatment. Intermittent hypoxia, achieved through a series of hypoxia and reoxygenation, was also studied in some of the selected articles [8,11,20,23].

In terms of which data sets from each study are reported in this article, only findings from parameters that represent osteoclast differentiation and osteoclast resorption activity are discussed. Osteoclast differentiation in vitro is assessed from the total number of multinucleated cells expressing osteoclast markers such as TRAP and VNR. Generally, osteoclast differentiation in vivo could be assessed from the number of osteoclasts using basic hematoxylin and eosin (H&E) staining (static histomorphometry) and TRAP staining on bone. Increased osteoclast formation in vitro and in vivo could also be characterized by elevated mRNA expression of osteoclast markers in the corresponding tissues. Elevated expression of osteoclast differentiation factors such as RANKL was not explicitly regarded as representing increased osteoclastogenesis since high levels of these proteins could also be observed in other conditions like early rheumatoid arthritis. Parameters on osteoclast activity in in vivo studies include the total bone resorption in the structural histomorphometry data such as erosion surface and bone volume. As for the in vitro studies, data from various resorption assays such as pit resorption assay and toluidine blue staining were used to represent osteoclast activity. Expression of bone degrading enzymes TRAP and cathepsin K as well as levels of systemic bone resorption marker CTX-1 are also used to indicate osteoclast bone resorption [8,20].

Comparison between all 16 in vitro studies (Tables S1–S3) found contradictions in findings in the impact of hypoxia on osteoclast formation. For instance, even though the studies by Gorissen et al. and Utting et al. [4,7] used the same in vitro model (human PBMC-derived osteoclasts), they obtained very different findings to each other. One explanation for this difference in findings is due to the different approaches in identifying osteoclasts used by those corresponding studies. Utting et al. [7] counted double nucleated TRAP-positive cells as osteoclasts; however, that was not the case in Gorissen et al. [4]. The differences in findings on the impact of hypoxia towards osteoclast differentiation between studies that used similar cell models might also be attributed to the used levels of O_2_ in the hypoxia treatment group. This can be seen in the study by Knowles and Athanasou [8] which demonstrated that the number of osteoclasts increased in 8% O_2_, but decreased in 2% and 0.1% O_2_. Findings from a recent study [30] indicated that the osteoclast differentiation process requires aerobic mitochondrial respiration. Different levels of O_2_ produced different numbers and sizes of osteoclasts [15]. Nomura et al. [15] also found that the timing of hypoxic treatment during the duration of osteoclast differentiation could also influence the total number of osteoclasts formed. The contradictory findings may also be attributed to various hypoxic conditions used by the investigators. Even though the studies described the hypoxic condition as a continuous low level of O_2_, it is difficult to constantly maintain the level of O_2_ throughout the experiments, including during the change of culture media. It is possible that constant hypoxia at <2% O_2_ inhibits osteoclast formation and activity, while hypoxia/ reoxygenation schedule enhances osteoclast activation [8].

Studies by Knowles et al. [8] and Muzylak et al. [9] reported that hypoxia stimulated osteoclast activity but not osteoclast differentiation. Their results suggested that hypoxia enhanced the bone resorption per unit osteoclasts, particularly at the 2% O_2_. This may also suggest that osteoclasts could resorb bone well in the absence of or minimal O_2_. Findings from a different study that was also on PBMC-derived osteoclasts indicated that the osteoclast bone resorption is more involved in the glycolytic pathway (that does not require O_2_); meanwhile, the osteoclast differentiation process is involved in aerobic respiration (which requires O_2_) [31]. A study demonstrated that matured or differentiated human monocyte-derived macrophages (which share a common progenitor lineage to osteoclasts) were also found to be equipped with enhanced glycolysis, probably to allow the cells to survive in inflammation sites and tumor microenvironment [32]. It was also suggested that human monocyte-derived macrophages had higher survivability than other cells in an anaerobic environment [32]. It was interesting to note that under extremely low O_2_ levels (0.2%), Knowles and Athanasou [8] reported a reduction in total resorption as a result of osteoclasts undergoing apoptosis as characterized by the trypan blue uptake and microscopic view of nuclei condensation. Further studies, such as assessing the expression of pro-apoptotic markers, need to be carried out to support the finding.

There were some thoughts that hypoxia promotes multinucleation during osteoclastogenesis, and this was suggested by Muzylak et al. [9], which was later supported by finding from another study reviewed here [7]. It was noted that hypoxia (2% O_2_) resulted in an increased number of nuclei per osteoclast by two folds [7]. Utting et al. suggested that hypoxia might increase bone resorption by increasing osteoclast size formed during osteoclastogenesis [7]. It is widely believed that the nucleation state of osteoclasts is an indicator of readiness of the osteoclasts in performing their bone resorptive function and capacity [33]. This may imply that hypoxia stimulated bone resorption possibly by enhancing the osteoclast bone resorption capacity, not only through promoting osteoclast differentiation as suggested by the majority of literature reviewed here.

It was interesting to note the finding in the study by Wang et al. (2016), in which micro-CT scanning revealed a reduction in the bone structure, bone mineral density, bone mineral content, bone volume, and trabecular number of rats following hypoxia (pO_2_ = 84 mmHg) in ovariectomized but not in normal rats [20]. This could suggest a possible involvement of estrogen in masking the effect of hypoxia on reducing those aforementioned bone structural parameters. It has been suggested that estrogen could suppress osteoclastogenesis, based on findings from previous studies, demonstrating an increased number of osteoclasts following estrogen deficiency in ovariectomized rats [34,35]. There was also a possibility that the findings of no significant decline in bone structure in normal hypoxic-treated rats as reported by Wang et al. (2016) was due to a short hypoxic treatment period. An earlier study demonstrated that normal rats staying in a long period of hypoxic condition (22–23 h per day for 42 days) had femur with lower bone mass and strength and stiffness [36].

As indicated earlier, several in vitro studies reviewed here also demonstrated that the hypoxia-induced osteoclast formation required permissive levels of exogenous RANKL and MCSF [5,7,19]. Even though Srinivasan et al. [14] demonstrated a higher rate of osteoclastogenesis following the addition of exogenous RANKL and MCSF, the finding was not statistically supported. While the addition of RANKL appeared to give an additive effect to hypoxia in promoting osteoclastogenesis of RAW264.7 (increase by about four-fold), data by Utting et al. [7] appeared to suggest RANKL and MCSF be essential in mediating osteoclastogenesis, regardless of normoxia or hypoxia, as this could be seen from the failure of human PBMC to differentiate into osteoclasts in the absence of RANKL and MCSF. Literature suggests that hypoxia could also promote osteoclastogenesis by regulating the expression of OPG and RANKL in osteoblasts [37] and osteocytes [38]. The significantly high mRNA expression of RANKL, as seen in the study by Takemori et al. [24], may indicate the pathological changes in breast cancer cells in hypoxic conditions (3% O_2_). The expression of this ligand may explain the occurrence of osteolysis in breast cancer to bone metastasis patients.

**Figure 2:**
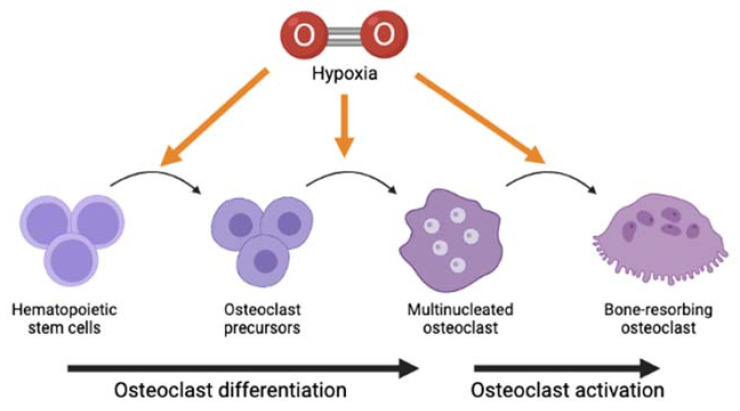
Graphical representation of possible stage(s) in osteoclast lifespan that hypoxia could affect, even though whether it either stimulates or suppresses the differentiation and activation of osteoclasts to resorb bone remains inconclusive.

Findings from all studies in this review provide us with some ideas on the possible intracellular signaling pathways that mediate the modulation of hypoxia on osteoclast differentiation and bone-resorbing function. Findings by Sun et al. [11], Zhao et al. [12], and Yu et al. [16] showed that hypoxia increased the expression of TRAF6, NFATc1, and c-Fos. Meanwhile, the study by Ma et al. [17], which reported contradictory findings, indicated that the suppression of osteoclastogenesis following hypoxia coincided with the lower gene expression of NFATc1 and c-Fos. The study also suggested that the hypoxia-inhibited osteoclastogenesis could be mediated by MAPK, IkBα, and JNK pathways. A previous study indicated that c-Fos-related protein Fra-2 controls osteoclast survival and size [39], suggesting the role of the c-Fos/AP-1-mediated pathway in response to extreme conditions like hypoxia. Collectively, all these findings may indicate that the process of hypoxia-modulated osteoclastogenesis is mediated by activation of these intracellular mediators and pathways. The changes in the expression of these genes suggest that hypoxia may modulate osteoclasts at multiple stages and aspects of osteoclasts biology, ranging from survival of osteoclast precursors [32], multinucleation process [6], and up to activation of mature osteoclasts to resorb bone [5,6] (Figure 2).

There are several limitations identified throughout this review. There is no gold standard level of O_2_ to define tissue hypoxia in vitro. This study identified some disagreements in findings regarding the effect of low O_2_ levels on osteoclast formation and activity. A meta-analysis on this subject matter would be an example of a future study that could be carried out. Future studies should include more research investigating the pathway or mechanism underlying the hypoxia-modulated osteoclast formation and activity. Concerning hypoxia, it would be interesting to conduct a systematic review or meta-analysis on the biology of osteoclasts in the context of either aerobic or anaerobic respiration. To further verify the requirement of RANKL and MCSF in hypoxia-induced osteoclast differentiation and activity in vitro, it would be interesting to study the impact of hypoxia in an osteoclast-osteoblast co-culture system [40] in the future. Since the majority of the findings reviewed here indicated that hypoxia resulted in more osteoclastogenesis, it may be suggested that hyperbaric oxygen therapy [41], aerobic exercises [42], and even pharmacological approach like HIF inhibitors such as 2-methoxyestradiol (2ME) and apigenin [43] could possibly be good therapeutic strategies for combating hypoxia-associated pathological bone loss like rheumatoid arthritis and osteoporosis.

## 4. Materials and Methods

A systematic review of the literature was conducted on the related studies of the effect of hypoxia on osteoclast formation and resorption. The search was performed using PUBMED MEDLINE and SCOPUS databases with articles published from 1946 to September 2020. The search strategy involved a combination of two set keywords: [hypoxi* OR aerobic* OR anaerobic*] AND [osteoclast*].

The search results were limited to original research articles published in the English language. Non-original research articles such as non-primary studies, reviews, news, proceedings, or editorials were excluded. The inclusion criteria for screening were articles related to the effect of hypoxia on osteoclast formation (osteogenesis or differentiation) or activity (resorption) using in vitro, in vivo, or human subjects.

Articles from both databases were gathered and screened independently in three phases by two reviewers. First was the title screening: any article with a title that did not match the inclusion criteria or was a duplicate was excluded. The second was abstract screening: abstracts of the remaining articles were screened and articles that did not meet the inclusion criteria were excluded. The third was content screening: full text of the remaining articles was read thoroughly to exclude articles that did not meet the inclusion criteria. The two reviewers must agree on those articles included before data extraction. Any dissimilar opinion was resolved through discussion with the third reviewer. In order to systematically collect the data, the data extraction form was standardized to include study characteristics as follows: (1) type of study; (2) sample of the study; (3) condition of the hypoxia; (4) methods used in the study; and (5) results of the study. Only data related to the direct effect of hypoxia on the formation and activity of osteoclasts were extracted from these studies.

## 5. Conclusions

In summary, even though data from the majority of literature reviewed indicated that low levels of O_2_ promoted osteoclast differentiation, there were several studies to indicate otherwise. There were also some studies indicating that osteoclastogenesis was suppressed at low levels of O_2_. Most of the studies reviewed here that assessed the osteoclast activity also found hypoxia to promote bone resorption carried out by osteoclasts. The contradictions in findings between studies might be attributed to differences in the experimental setup. Future studies should focus on the underlying mechanism of hypoxia-modulated osteoclastogenesis that will provide a better understanding, allowing the discovery of good therapeutic targets for hypoxia-associated pathological bone loss.

## Data Availability

Third party data. Restrictions apply to the availability of these data. Data were obtained from existing literature and reviewed articles are accessible from the PubMed and Scopus search engines.

## Supplementary Materials

The following are available online at https://www.mdpi.com/article/10.3390/ijms221810146/s1.
