# Supplementary material for: Impacts of Hypoxia on Osteoclast Formation and Activity: Systematic Review
Source: Int J Mol Sci. 2021 Sep 20;22(18):10146. doi: 10.3390/ijms221810146 (PMC8467526; doi:10.3390/ijms221810146)
Supplement: Supplementary file 1 [file ijms-22-10146-s001.zip › Table S1.pdf]

Table S1: Studies on impacts of hypoxia on the differentiation and activity of PBMC-derived osteoclasts and osteoclast-like giant cells

Study	Author	Type of Study & Sample	Hypoxic Conditions	Methods	Results
1.	Gorrisen *et al.* (2018)	*in vitro;*<br>CD14+ monocytes from human peripheral blood mononuclear cells (PBMCs) were cultured with M-CSF and RANKL supplementation.	5% O_2 throughout the experiment (24 h, 1, 2 and 3 weeks)	a. Osteoclasts were identified by TRAP staining.<br>b. Resorption was measured through bisphosphonate fluorescence visualized with confocal microscopy.<br>c. Gene expression of *CA II*, *ITGB3*, *TRAP*, *CatK* and *DCSTAMP* was measured by RT-qPCR.	a. Hypoxia reduced the intensity of TRAP positive osteoclasts..<br>b. Hypoxia delayed TRAP-positive osteoclast formation.<br>c. Hypoxia decreased mRNA levels of *CA II*, ITGB3, *TRAP*, CatK and *DCSTAMP*.<br>d. Hypoxia increased single and double nucleated cells but not multinucleated.<br>e. Total bone resorption capacity was not affected by hypoxia exposure over the 21 days.<br>f. Hypoxia decreased senescence marker p21and SA-β-gal expression
2.	Murata *et al.* (2017)	*in vitro;*<br>CD14+ monocytes from human peripheral blood mononuclear cells (PBMCs) were cultured with M-CSF and RANKL supplementation.	2% O_2 for 3 h before RANKL was added.	a. Osteoclasts were identified by TRAP staining.<br>b. Resorption was measured through the Osteo Assay Surface plate method.<br>c. Gene expression of *ITGB3,* cathepsin K *CatK* and *CTR* was measured by RT-qPCR.	a. Hypoxia increased the number of TRAP-positive osteoclasts.<br>b. Resorption was higher in cultures under hypoxia<br>c. Hypoxia decreased *ITGB3*, *CatK* and *CTR* expression
3.	Hulley *et al.* (2017)	*in vitro;*<br>CD14+ monocytes of PBMCs from leukocyte cones (NHS Blood and Transplant) were cultured with M-CSF and RANKL supplementation for 9 days.	2% O_2 for 24 h.	a. Osteoclasts were identified by TRAP staining.<br>b. VNR-positive osteoclast was detected by immunocytochemistry.<br>c. Resorption was measured through toluidine blue staining method.	a. Number of VNR-positive osteoclasts was not different between hypoxia and normoxia on day 2-3, 4-5, 6-7 and 8-9 of differentiation.<br>b. Resorption was higher in cultures under hypoxia on day 4-5, 6-7 and 8-9 of differentiation.
4.	Utting *et*	*in vitro;*	Hypoxia: 0.2-12%	a. Osteoclasts were identified by	a. Hypoxia increased the osteoclast number

	al. (2010)	Mononuclear cells were isolated from healthy male and female human PBMCs aged 20-50. The mononuclear cells obtained were cultured with M-CSF supplementation.	O_2 for 10 days.	TRAP staining.	b. Hypoxia increased the number of nuclei per osteoclast<br>c. Resorption was higher ~~resorption pit formation per osteoclast and total amount of resorption were higher in cells under~~ under hypoxia (2% O_2).
5.	Knowles *et al*. (2009)	*in vitro;*<br>Osteoclast-like giant cells of giant cell tumor of bone (GCTB) were isolated from tumor tissue and used as a model of mature human osteoclast.	Hypoxia: 8, 5, 2 and 0.1% O_2 for 24 h.	a. Resorption area was visualized using toluidine blue staining.<br>b. CTX-1 level in culture media was measured by ELISA.<br>c. TRAP and CatK activities were measured by spectrometry.<br>d. VNR was visualized by immunohistochemistry.	a. Resorption area was higher in cultures under 5 and 2% O_2, while was lower in 0.1% O_2 compared to normoxia.
		in vitro;<br>Mononuclear cells derived from PBMCs were cultured with M-CSF and RANKL supplementation.	Hypoxia: 8, 5, 2 and 0.1% O_2 for 24 or 72 h.<br>a. 24 h 2% O_2<br>b. 24 h 2% O_2 + 24 h normoxia<br>c. 72 h hx/rx (reoxygenation) : 3X of 1 h normoxia + 23 h 2% O_2<br>d. 7 days: 2% O_2 on days 14-21<br>e. 21 days hx/rx: 21X of 1 h normoxia + 23 h 2% O_2		a. Resorption area and TRAP activity were higher in cultures under 5 and 2% O_2 for 24 h.<br>b. The mRNA level of CTX-1 release in media and CatK was higher in 2% O_2 for 24 h.<br>c. Number of VNR-positive cells was higher in cultures under 8% O_2, while was lower in cultures under 2 and 0.1% O_2 for both 24 and 72 h.<br>b. Number of toluidine blue-positive cells was lower in cultures under 0.1% O_2 for 24 h, 2 and 0.1% O_2 for 72 h.<br>a. Number of toluidine blue-positive cells was lower in cultures under 2% O2 on days 14-21, while cell number was not different between other conditions and normoxia.

6.	Muzylak *et al*. (2006)	*in vitro;* PBMCs isolated from healthy feline blood of 2 years old. Cells were cultured with M-CSF and RANKL supplementation.	Hypoxia: 12 or 2% O_2 on days 1-14 or 11-14.	a. Osteoclasts were identified by TRAP staining.<br>b. Resorption was visualized by biotin-conjugated wheat germ agglutination lectin staining after reacted with TRITC-streptavidin.	a. Hypoxia lowered the number of TRAP-positive osteoclasts when exposed to 12 and 2% O_2,<br>b. The size of each osteoclast was bigger in 2% O_2 compared to 20% O_2 exposure on days 1-14.<br>c. The size of TRAP-positive osteoclasts was bigger while their number remained unchanged in cultures exposed to 2% O_2 on 11-14 days.<br>d. Resorption percentage was higher in cultures exposed to 12 and 2% O_2 on days 1-14 and 11-14.

Note: Normoxia or reoxygenation (rx): 20-21% O_2. Abbreviations: Oxygen (O_2), Quantitative reverse transcriptase polymerase chain reaction (RT-qPCR), messenger RNA (mRNA), Tetramethylrhodamine (TRITC), Enzyme linked immunosorbent assay (ELISA), Tartrate-resistant acid phosphatase (TRAP), Carbonic anhydrase II (CA II), Integrin subunit beta-3 (ITGB3), Cathepsin K (CatK) and Dendritic cell-specific transmembrane protein (DCSTAMP), Calcitonin receptor (*CTR*), Vitronectin receptor (VNR) & hypoxia (hx).
