# Supplementary material for: Impacts of Hypoxia on Osteoclast Formation and Activity: Systematic Review
Source: Int J Mol Sci. 2021 Sep 20;22(18):10146. doi: 10.3390/ijms221810146 (PMC8467526; doi:10.3390/ijms221810146)
Supplement: Supplementary file 1 [file ijms-22-10146-s001.zip › Table S2.pdf]

**Table S2:** Studies on impacts of hypoxia on differentiation and activity of osteoclasts derived from RAW264.7 cell line *in vitro*

| Study | Author | Type of Study & Sample | Hypoxic Conditions | Methods | Results |
|---|---|---|---|---|---|
| 1. | Tang *et al.* (2019) | *in vitro;*<br>RAW264.7 cells were cultured with RANKL supplementation | 2% $O_2$ for 24 h | a. Osteoclasts were identified by TRAP staining.<br>b. Resorption was measured through toluidine blue staining *in vivo* and *in vitro*.<br>c. Gene expression of *TRAP*, *CTSK* and *MMP9* were measured by RT-qPCR.<br>d. Phalloidin staining *in vitro* with areas of phalloidin-positive cells were reported. | a. Hypoxia increased the number of osteoclasts with 6-10 nuclei significantly<br>b. Hypoxia increased TRAP, CatK and MMP-9 mRNA expression.<br>c. Hypoxia increased TRAP-positive osteoclasts, resorption area (from toluidine blue staining) and relative phalloidin areas. |
| 2. | Ma *et al.* (2018) | *in vitro;*<br>RAW264.7 cells were cultured without RANKL supplementation. | 1% $O_2$ for 3 and 5 days for 24 hours | a. Osteoclasts were identified by TRAP staining.<br>b. Gene expression of RANK, CatK, TRAP, and NFATc1 were measured by RT-qPCR.<br>c. TRAP, CatK, RANK, p-JNK, JNK, p-ERK and ERK, p-P-38, P-38, IKB, p-IKB, NFATc1 protein expression through Western blotting | a. Number of TRAP-positive multinucleated cells was lower in cultures under 1 % $O_2$ for both cell types.<br>b. The size of multinucleated cells was smaller in cultures under 1 % O'2 For both cell types.<br>c. Resorption area was smaller in cultures under 1% $O_2$<br>d. mRNA expression RANK, CatK, TRAP, and NFATc1 was lower in cultures under 1% $O_2$<br>e. The phosphorylation of JNK and IKB in RAW264.7 cells did not reduce in hypoxia 1% $O_2$<br>f. The mRNA and protein expression of NFATc1 is lower in hypoxia 1% $O_2$ for both cell types. |
| 3. | Sun *et al.* | *in vitro;* | 0.3% $O_2$ for 24 | a. Osteoclasts were identified by | a. Number of TRAP-positive |

| | | | | | |
|---|---|---|---|---|---|
| | (2015) | RAW264.7 cells were cultured with RANKL and M-CSF | hours, followed by normoxia for 6 days. | TRAP staining.<br>b. Gene expression of TRAF6, NFATC1, CatK, cFOS and MMM9 were measured by RT-qPCR. | multinucleated cells was higher under hypoxia.<br>b. Expression levels of TRAF6, NFATc1, CatK, cFOS and MMM9 were upregulated in hypoxia compared to normoxia |
| 4. | Zhao *et al.* (2011) | *in vitro;*<br>RAW264.7 cells were cultured without RANKL supplementation | 0.2% $O_2$ for 2-24 h. | a. Osteoclasts were identified by TRAP staining.<br>b. Gene expression of TRAP, cathepsin K (CatK), RANKL, matrix metalloproteinase 9 (MMP9) and NFATc1 were measured by RT-qPCR. | a. Number of TRAP-positive multinucleated cells was higher in cultures under hypoxia for 4, 6, 8, 12 and 24 h.<br>b. The mRNA level of osteoclastogenesis-related markers was higher in cultures under hypoxia for:<br>i. 2 h: NFATc1<br>ii. 4 h: MMP9 and NFTAc1<br>iii. 6, 8 and 12 h: TRAP, CatK, RANKL, MMP9 and NFATc1.<br>iv. 24 h: TRAP, Catk, and MMP9 |
| 5. | Leger *et al.* (2010) | RAW264.7 cells were cultured with RANKL supplementation | 5% $O_2$ for 4 and 6 days. | a. Osteoclasts were identified by TRAP staining. | a. Number of TRAP-positive multinucleated cells was higher in cultures under hypoxia for 4 days, while there was no difference for 6 days. |
| 6. | Srinivasan *et al.* (2007) | *in vitro;*<br>RAW264.7 cells were cultured with and without RANKL supplementation | Hypoxia:<br>RT-qPCR: 0.5% $O_2$ for 10 h<br>TRAP staining: 5% $O_2$ for 5 or 6 days | a. Osteoclasts were identified by TRAP staining.<br>b. Gene expression of CTR, CatK and TRAP were measured by RT-qPCR | a. Number of TRAP-positive multinucleated cells in cultures under hypoxia. However there are no indication of statistical significance<br>b. Level of CTR mRNA was higher in cultures under hypoxia without RANKL, while there was no difference between |

| | | | | | |
|---|---|---|---|---|---|
| | | | | | hypoxic and normoxic conditions with RANKL. Level of CatK and TRAP mRNA was higher in hypoxia with RANKL, while there was no difference between hypoxia and normoxia without RANKL. However there are no indication of statistical significance |

Note: Normoxia or reoxygenation: 20-21% $O_2$. Terms & Abbreviations: Oxygen ($O_2$), Quantitative reverse transcriptase polymerase chain reaction (RT-qPCR), messenger RNA (mRNA), Tartrate-resistant acid phosphatase (TRAP), Cathepsin K (CatK), Calcitonin receptor (CTR), Vitronectin receptor (VNR), Matrix metallopeptidase 9 (MMP9), Nuclear factor of activated T cell 1 (NFATc1), Receptor activator of NF-κββ ligand (RANKL), Janus kinase (JNK), Tumor necrosis factor receptor-associated factor 6 (TRAF6), Inhibitor Of Nuclear Factor Kappa-B Kinase Subunit Beta (IKB), Mitogen-activated protein kinase 11 (P38) & Extracellular signal-regulated kinase (ERK)
