# Supplementary material for: Impacts of Hypoxia on Osteoclast Formation and Activity: Systematic Review
Source: Int J Mol Sci. 2021 Sep 20;22(18):10146. doi: 10.3390/ijms221810146 (PMC8467526; doi:10.3390/ijms221810146)
Supplement: Supplementary file 1 [file ijms-22-10146-s001.zip › Table S3.pdf]

Table S3: Studies on impacts of hypoxia on the differentiation and activity of osteoclasts derived from bone marrow cells *in vitro*

Study	Author	Type of Study & Sample	Hypoxic Conditions	Outcome Parameters	Findings
1.	Yu et al. (2020)	*in vitro*; Murine bone-marrow-derived monocytes (BMMs) flushed from 3 w.o. C57BL/6 mice, grown until 5 days and were cultured with M-CSF and RANKL	2% O_2	a. Osteoclast was detected by TRAP staining. b. Resorption was measured on Corning Osteo Assay Surface plates using Image J. c. NFATc1, cathepsin K (CatK), c-Fos protein expression through Western blotting	a. Hypoxia increased TRAP positive osteoclasts. b. Total resorption area was higher under hypoxic conditions. c. Hypoxia increased the protein expression of NFATc1, CatK and c-Fos.
2.	Ma et al. (2018)	*in vitro;* Murine BMMs were obtained from the tibiae of 6-8 w.o C57BL/6 mice, were cultured with M-CSF and RANKL supplementation for 3 days before hypoxia treatment ~~(for outcome parameter TRAP staining).~~	1% O_2 for 7 days for TRAP staining. For the resorption assay, 1% O_2 for 15 days	a. Osteoclasts were identified by TRAP staining. b. Resorption was measured by toluidine blue staining. c. Gene expression of TRAP RANK, CatK, TRAP, and NFATc1 was measured by RT-qPCR.	a. Number of TRAP-positive multinucleated cells was lower in 1 % O_2 for both cell types. b. The size of multinucleated cells was smaller in cultures under 1 % O_2 for both cell types. c. Resorption area was smaller in cultures under 1% O_2 d. The mRNA expression of RANK, CatK, TRAP, and NFATc1 was lower in cultures under 1% O_2 e. The mRNA of NFATc1 is lower in hypoxia 1% O_2 for both cell types.
3.	Nomura *et al.* (2015)	*in vitro;* Human bone marrow cells (BMCs) from donors who underwent total hip arthroplasty for osteoarthritis were cultured with M-CSF and RANKL supplementation.	Hypoxia: 10, 5, 3 or 1% O_2 for 21 days. Time course approaches: a. Day 1 to 21 - Normoxia b. Day1 to 7 days - Normoxia + 14 days - 5% O_2 c. Day1 to 14 days - Normoxia + 7 days -	a. Osteoclasts were identified by TRAP staining.	a. Number of TRAP-positive multinucleated osteoclast cells formed was not significantly more under 1% O_2 hypoxia. 3-10% O_2 increased number of TRAP-positive cells b. Hypoxic (5% O_2) exposure during the early and longer culture time points enhanced differentiation of BMCs into OCs

			$5\%\ O_2$<br>d. Day1 to 7 days - $5\%\ O_2$ + 14 days - Normoxia<br>e. Day1 to 14 - $5\%\ O_2$ + 7 days - Normoxia<br>f. $5\%\ O_2$ for 21 days		c. Hypoxic ($5\%\ O_2$) exposure during the early and longer culture time points increased the number of pre-osteoclasts and facilitated differentiation of pre-osteoclasts into multinucleated osteoclasts.

4.	Fukuoka *et al.* (2005)	*in vitro;*<br>Murine bone marrow cells (BMCs) were obtained from the tibiae of adult ddY mice aged 5-7 weeks old and were cultured with M-CSF and RANKL supplementation.	Hypoxia: 10, 5, 1 or $0\%\ O_2$ for 6 days.	a. Osteoclasts were identified by TRAP staining.	a. Number of TRAP-positive multinucleated cells TRAP-positive multinucleated cells was higher under 5 and $1\%\ O_2$
5.	Arnett *et al.* (2003)	*in vitro;*<br>Murine bone marrow cells (BMCs) were derived from long bone of 8 weeks old MF1 mice and were cultured with M-CSF and RANKL supplementation.	12, 5 and $2\%\ O_2$ for 7 days.	a. Osteoclasts were identified by TRAP staining.<br>b. Resorption was measured by toluidine blue staining.	a. Number of TRAP-positive multinucleated cells was higher in cultures under 5 and 2 $\%\ O_2$.<br>b. Resorption area was bigger in cultures under 12, 5 and $2\%\ O_2$.
			12, 5, 2, 1 and 0.2% O_2 for 13 days		a. Number of TRAP-positive multinucleated cells and the resorption area were higher in cultures under 5, 2, 1 and $0.2\%\ O_2$.
		in vivo;<br>Mature rat osteoclasts	12, 5, 2, 1 and 0.2% O_2 for 26 h.		a. Number of TRAP-positive multinucleated cells was lower in

		were isolated from minced long bones of neonatal Sprague Dawley rats of 2 days old.			cultures under 2, 1 and 0.2% O_2.<br>b. Resorption area was bigger in cultures under 0.2% O_2.

Note: Normoxia or reoxygenation: 20-21% O_2. Abbreviations: Oxygen (O_2), Quantitative reverse transcriptase polymerase chain reaction (RT-qPCR), Tartrate-resistant acid phosphatase (TRAP), Integrin subunit beta-3 (ITGB3), Cathepsin K (CatK), Carboxy-terminal cross-linked telopeptide of type 1 collagen (CTX-1), Matrix metallopeptidase 9 (MMP9), Nuclear factor of activated T cell 1 (NFATc1), Receptor activator of NF-κββ ligand (RANKL)
