# Supplementary material for: Impacts of Hypoxia on Osteoclast Formation and Activity: Systematic Review
Source: Int J Mol Sci. 2021 Sep 20;22(18):10146. doi: 10.3390/ijms221810146 (PMC8467526; doi:10.3390/ijms221810146)
Supplement: Supplementary file 1 [file ijms-22-10146-s001.zip › Table S4.pdf]

Table S4: Summary of studies on impacts of hypoxia on osteoclast formation and activity *in vivo*

Study	Author	Type of Study & Sample	Hypoxic Conditions	Methods	Findings
1.	Durand et al. (2019)	*in vivo;*<br>10-week-old male C57BL/6J mice	4 days of hypobaric hypoxia FiO2 = 10%	a. Osteoclast number/bone surface (Oc. N/ BS) was measured by bone static histomorphometry.	a. Hypoxia drastically increased Oc.N/ BS by 2 fold.<br>b. Hypoxia did not affect bone volume (BV/TV)
2.	Takemori et al. (2018)	*in vivo*:<br>5 w.o. female BALB/c mice in which cell line was intremedullary implanted into proximal epiphysis	CO_2 hydrogel was applied at the tissue where the cells were implanted 4 weeks before.	a. Osteoclast number on CO_2 treated tibiae was detected by tartate-resistant acid phosphatase (TRAP) staining.<br>b. Micro-CT for bone volume	a. Transcutaneous application of CO_2 hydrogel decreased TRAP-positive cells on treated tibiae.
3.	Wang *et al.* (2016)	*in vivo;*<br>Normal or ovariectomized (OVX) adult female Sprague Dawley rats of 12 weeks old	Intermittent hypobaric hypoxia was induced at 4 weeks after ovariectomy procedure. Hypoxic condition was performed using experimental high-altitude imitation: rats were housed in a chamber with reduced pressure equivalent to an altitude of 5000 m (P_B=404 mmHg; P_{O2}=84 mmHg) for 4 h once daily for 14 consecutive days.	a. Serum level of C-telopeptide of collagen (CTX) as bone resorption marker was measured by ELISA.<br>b. Osteoclasts in the femur were identified using TRAP staining.	a. Osteoclast number and serum CTX-I level were higher in OVX rats that were exposed to intermittent hypobaric hypoxia.<br>b. No change in between normal rats under hypoxia or normaxia.
4.	Dalle Carbonare *et al.* (2015)	*in vivo;*<br>Humanized mouse model for sickle cell disease (SCD: *Hba*$^{tm1(HBA)Tow}$ *Hbb*$^{tm2(HBG1,HBB^{*})Tow}$ SS) and	Hypoxia/reoxygenation (H/R) stress: hypoxia (8% O_2 for 10 h) followed by reoxygenation for 3 h	a. mRNA level of RANK in tibia bones was determined using RT-qPCR.<br>b. Osteoclast number/tissue area (N.Oc/TA) and erosion surface/bone surface (ES/BS)	a. mRNA level of RANK in bone was higher in both healthy and SCD mice under H/R stress compared with normaxia.<br>N.Oc/TA, ES/BS and serum CTX levels were higher only in SCD mice

		healthy control (*Hba*$^{tm1(HBA)Tow}$ *Hbb*$^{tm3(HBG1,HBB)Tow}$ AA) aged 16-18 weeks, male and female.		were measured by bone histomorphometry.<br>c. Serum CTX-1 level was measured by ELISA.	under H/R stress.

Note: Normoxia or reoxygenation: 20-21% O_2. Terms & Abbreviations: Oxygen (O_2), Quantitative reverse transcriptase polymerase chain reaction (RT-qPCR), messenger RNA (mRNA), Enzyme linked immunosorbent assay (ELISA), Number of osteoclasts over Total bone area (N.Oc/TA), Erosion surface over bone surface (ES/BS), Tartrate-resistant acid phosphatase (TRAP) & Carboxy-terminal cross-linked telopeptide of type 1 collagen (CTX-1).
